# Bacilladnaviridae: refined taxonomy and new insights into the biology and evolution of diatom-infecting DNA viruses

**DOI:** 10.1099/jgv.0.002084

**Published:** 2025-03-12

**Authors:** Arvind Varsani, Joy M. Custer, Ilaria N. Cobb, Ciara Harding, Courtney L. Collins, Crystal Suazo, Joshua Schreck, Rafaela S. Fontenele, Daisy Stainton, Anisha Dayaram, Sharyn Goldstein, Darius Kazlauskas, Simona Kraberger, Mart Krupovic

**Affiliations:** 1 The Biodesign Center for Fundamental and Applied Microbiomics, Center for Evolution and Medicine, School of Life Sciences, Arizona State University, Tempe, AZ 85287, USA; 2 Structural Biology Research Unit, Department of Integrative Biomedical Sciences, University of Cape Town, 7925, Cape Town, South Africa; 3 School of Biological Sciences, University of Canterbury, Christchurch, New Zealand; 4 Institute of Neurophysiology, Charité Universitätsmedizin Berlin, 10117 Berlin, Germany; 5 Institute of Biotechnology, Vilnius University, Saulėtekio av. 7, Vilnius 10257, Lithuania; 6 Institut Pasteur, Université Paris Cité, CNRS UMR6047, Archaeal Virology Unit, Paris, France

**Keywords:** algal viruses, *Bacilladnaviridae*, *Cressdnaviricota*, diatoms, single jelly-roll capsid proteins, ssDNA viruses

## Abstract

Bacilladnaviruses are single-stranded DNA viruses that infect diatoms that, so far, have been primarily identified in marine organisms and environments. Using a viral metagenomics approach, we discovered 13 novel bacilladnaviruses originating from samples of mud-flat snail (*Amphibola crenata*; *n*=3 genomes) and benthic sediments (*n*=10 genomes) collected from the Avon-Heathcote Estuary in New Zealand. Comparative genomics and phylogenetic analysis of the new bacilladnavirus sequences in the context of the previously classified members of the family helped refine and further expand the *Bacilladnaviridae* taxonomy. Here, based on the replication-associated protein phylogeny and pairwise identities, we established 4 new genera – *Aberdnavirus*, *Keisodnavirus*, *Puahadnavirus* and *Seawadnavirus* – and 13 new species within the family. Comparison of the bacilladnavirus capsid protein sequences suggests that the positively charged N-terminal region (R-arm) is required for encapsidation of the larger genomes, whereas the smaller bacilladnavirus genomes can be packaged in the absence of the R-arm subdomain. Furthermore, analysis of the bacilladnavirus genomes revealed that members of three genera encode a highly derived variant of a phospholipase A1, which is predicted to be involved in the lysis of the infected diatoms and/or facilitates the entry of the virions into the host cells. Collectively, our results allow refining of the taxonomy of bacilladnaviruses and provide new insights into the biology and evolution of this understudied group of diatom viruses.

## Introduction

Bacilladnaviruses are single-stranded DNA viruses in the phylum *Cressdnaviricota* (class *Arfiviricetes*, order *Baphyvirales*) [1], with genomes ranging from ~3.5 to 6 kb. Bacilladnavirus genomes encode at least four major ORFs, but only two of them can currently be assigned functions, namely the replication-associated protein (Rep) and capsid protein (CP). As all other members of the *Cressdnaviricota*, bacilladnaviruses appear to replicate by a rolling circle mechanism and encode a characteristic Rep protein with two domains, the N-terminal HUH superfamily endonuclease domain and the C-terminal superfamily 3 helicase domain [2]. The two domains display congruent phylogenies within the family, suggesting coevolution [3]. The CP of bacilladnaviruses is structurally most closely related to the CPs of positive-sense RNA viruses of the *Nodaviridae* family [2,4]. Notably, bacilladnaviruses are the only viruses in the *Cressdnaviricota* that have been shown to form T=3 icosahedral capsids [4], with all other members in this phylum having smaller T=1 capsids. Bacilladnaviruses cause lysis of their hosts, but the mechanism of cell lysis is unknown.

All bacilladnaviruses for which hosts have been experimentally determined infect diatoms, primarily *Haslea ostrearia* and members of the *Chaetoceros* genus [5,12]. Bacilladnaviruses have also been identified in mud-flat snail/tītiko (*Amphibola crenata*) [2] and red snapper (*Lutjanus* sp.) [13] tissues as well as in various marine/estuarine animal/environmental samples, such as ocean water [14] and estuarine benthic sediments [2], where they are also likely to infect diatoms.

The family *Bacilladnaviridae* was created in 2018 [15] and included three genera, namely *Diatodnavirus* (one species), *Kieseladnavirus* (one species) and *Protobacilladnavirus* (seven species) [16]. The genera were established based on the phylogeny of the HUH superfamily Rep, the most conserved protein encoded by bacilladnaviruses, with a 75% amino acid identity threshold chosen as a species demarcation criterion. Notably, the Rep sequences of bacilladnaviruses are highly divergent compared to those of other cressdnaviricots, resulting in the unstable position of the bacilladnavirus clade in global phylogenies [17]. Further exploration of the bacilladnavirus diversity is expected to help mitigate this situation.

Here, we describe the identification of 13 bacilladnavirus genomes from mud-flat snail (*n*=3) and benthic sediments (*n*=10), both collected from Avon-Heathcote Estuary (Ihutai) in New Zealand (Aotearoa). We further analyse these genomes with 17 other bacilladnavirus genomes available in GenBank to refine and update the taxonomy of the family *Bacilladnaviridae*.

## Methods

### Sample collection and identification of complete viral genomes

As part of a study to identify viruses in estuarine ecosystems, we collected ~20 individuals of mud-flat snail/tītiko and ~20 g of benthic sediments from the Avon-Heathcote estuary (Ihutai; location 43.5578 s, 172.7058 E) in July 2012. Samples were refrigerated upon collection. The estuary forms the confluence of two rivers (the Avon and Heathcote), which flow through the city of Christchurch, New Zealand (Aotearoa). The mud-flat snail samples were removed from the shells and washed in sterile distilled water, and the whole bodies were then pooled. The pooled mud-flat snail samples and the benthic sediments were homogenized separately in SM buffer [0.1 M NaCl, 50 mM Tris/HCl (pH 7.4), 10 mM MgSO_4_] at a ratio of 10 ml SM buffer to 5 g of tissue/benthic sediments. Cellular debris was pelleted by centrifuging (10 000 ***g*** for 10 min), and the supernatant was sequentially filtered through 0.45 µm and 0.2 µm syringe filters (Sartorius Stedim Biotech, Germany). The viral DNA was extracted from 200 µl of filtrate using the High Pure Viral Nucleic Acid Kit (Roche, USA). To enrich circular DNA, we used rolling circle amplification (RCA) using TempliPhi 2000 (GE Healthcare, USA).

The RCA products of the two samples were used to generate 170 bp insert libraries, which were sequenced on an Illumina HiSeq 2000 (Illumina, USA) platform at the Beijing Genomics Institute (Hong Kong). The raw reads (for a 2×91 nt) were trimmed with Trimmomatic v0.39 [18] and *de novo* assembled using metaSPAdes 3.14.1 [19]. The *de novo* assembled contigs were analysed against a viral RefSeq protein database of bacilladnavirus protein sequences using blastx [20]. All contigs with hits to bacilladnavirus protein sequences were checked for terminal redundancy to determine if they were circular. For the circular bacilladnavirus-like contigs (*n*=13), we used ORFfinder (https://www.ncbi.nlm.nih.gov/orffinder/) to identify and annotate ORFs.

### Viral genome sequence analysis

The full genome sequences of bacilladnaviruses available in GenBank (*n*=17) were downloaded and analysed together with the 13 newly identified ones from mud-flat snail/tītiko (*n*=3) and benthic sediments (*n*=10). The Rep and CP sequences were extracted, translated and assembled into datasets for further analysis. Rep and CP sequence datasets were aligned using MAFFT [21], and these alignments were used to infer maximum likelihood phylogenetic trees using PhyML 3.0 [22] with best-fit model rtREV+G+I determined using ProtTest 3 [23]. Representative sequences from the *Circoviridae* family were used to root the Rep phylogenetic tree. The CP phylogenetic tree was midpoint rooted. PLA1 sequences were aligned using PROMALS3D [24], and the alignments visualized using Jalview v2 [25]. All sequence pairwise identities were determined using SDT v1.2 [26].

### Identification of the bacilladnavirus PLA1 homologues

Sequences of hypothetical bacilladnavirus proteins (i.e. not CP or Rep) were analysed using HHsearch against the PFAM (Database of Protein Families), Protein Data Bank (PDB), Conserved Domain Database, and uniprot_sprot_vir70 databases [27]. The structures of representative bacilladnaviral PLA1 homologues were modelled using ColabFold v1.5.5 [28] ‘alphafold2_multimer_v3’ model with six recycles. Structures were searched against the PDB database using DALI [29]. Protein structures and structural models were visualized using UCSF ChimeraX v1.7.1 [30].

## Results and discussion

### Identification of new bacilladnavirus genomes

**Fig. 1.**
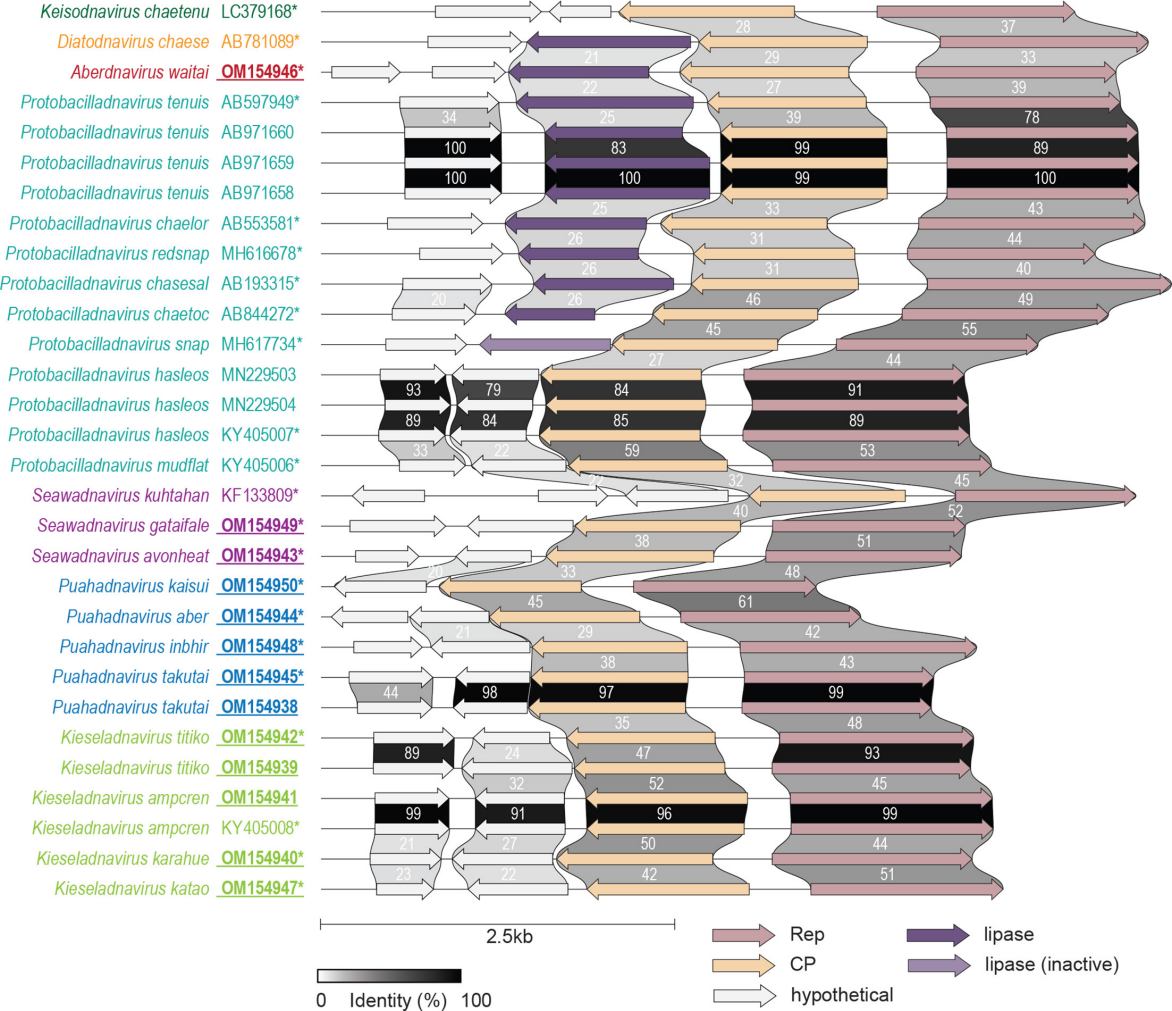
Genome organization of all the bacilladnaviruses (complete genomes) available in GenBank. Genome comparisons were generated using Clinker [67]. The species names and accession numbers are provided on the left. The genera that the viruses belong to are colour coded. The accession numbers of the new sequences identified from mud-flat snail and benthic sediments sampled from the Avon-Heathcote Estuary (Ihutai) in New Zealand are in bold font and underlined. An asterisk (*) next to the accession number denotes representative sequences for each species.

We identified 13 bacilladnavirus genomes that range in size from 3492 to 5608 nt (Fig. 1). Three of these were from mud-flat snail and ten from benthic sediments sampled from the Avon-Heathcote Estuary in New Zealand. It is most likely that all these bacilladnaviruses infect diatoms that have been indirectly sampled. Consistently, the mud-flat snail is a detritus or deposit feeder, which is known to feed on diatoms and other microbes [31]. Notably, the genome of avonheates virus Gas_1207 (OM154941) from mud-flat snail shares 99.6% genome-wide pairwise identity with Avon-Heathcote estuary associated bacilladnavirus AHEaBavV1 (KY405008), previously identified in benthic sediments at the same location [2]. The two viruses belong to the same species, further supporting that the mud-flat snail-associated viruses infect diatom hosts.

**Fig. 2.**
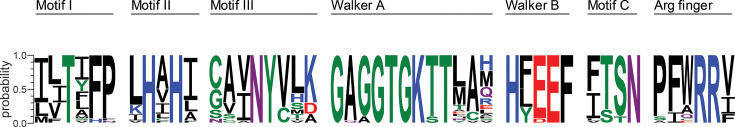
Sequence probability logos, generated using WebLogo 3 [68], of the motifs conserved in bacilladnavirus Reps. The rolling circle replication endonuclease domain includes motifs I, II and III, whereas the superfamily 3 helicase domain includes Walker A and B, motif C and the Arg finger.

The Rep sequences of bacilladnaviruses contain the three characteristic RCR motifs I, II and III [32,34] and the superfamily 3 helicase motifs Walker A, Walker B, motif C [35,36], and the Arg finger [17] (Fig. 2). The conserved motifs display signatures that distinguish bacilladnaviruses from other members of the phylum *Cressdnaviricota* [1]. A distinct feature of bacilladnaviruses is the FP and PF residues in the motif I and Arg finger, respectively, as well as the two glutamates in the Walker B motif, instead of the aspartates present in the Reps of most other cressdnaviricots.

### Classification and nomenclature of new bacilladnaviruses

**Fig. 3.**
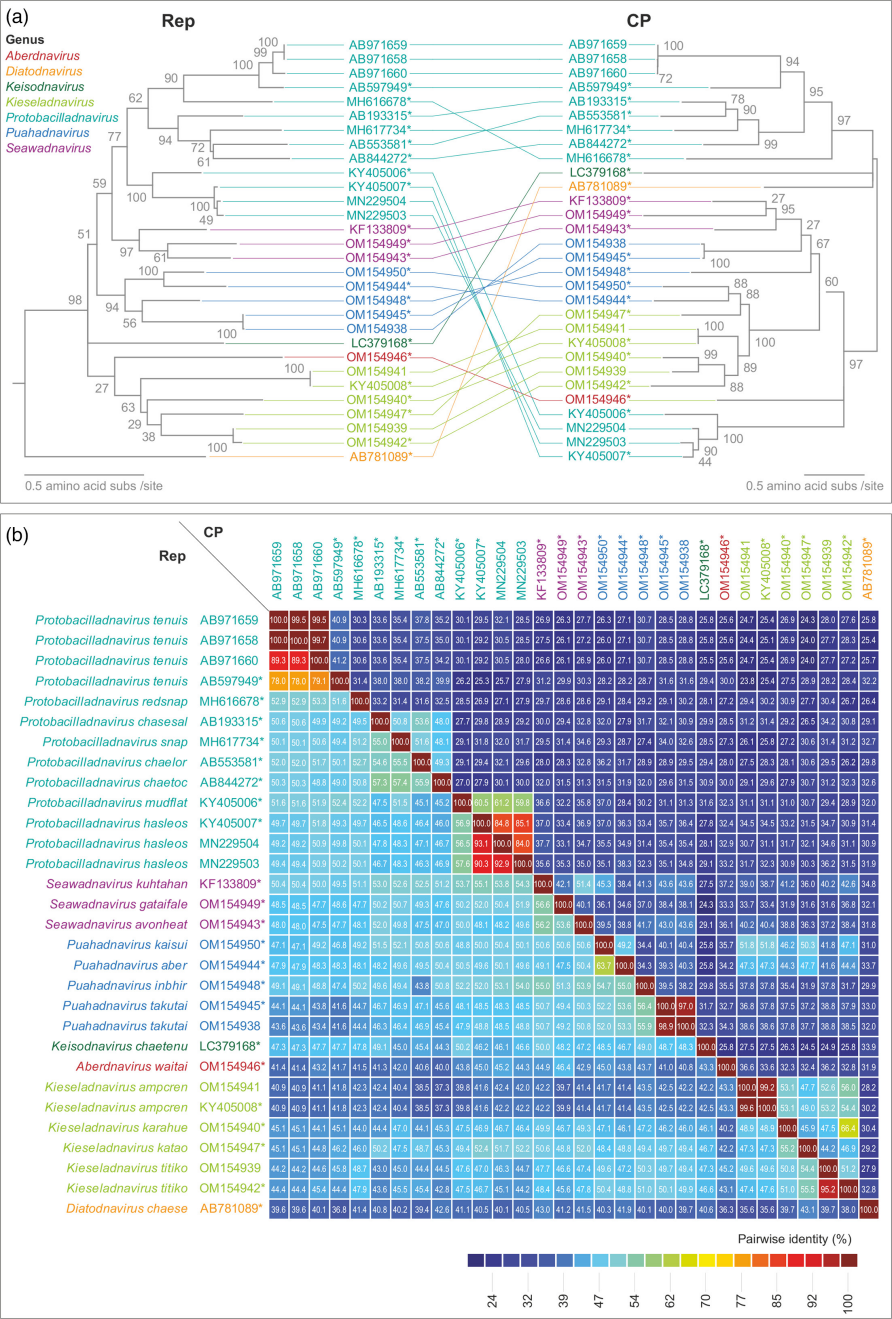
Evolutionary histories of Rep and CP coding genes within bacilladnavirus genomes. (a) Tanglegram of maximum likelihood phylogenetic trees of the Rep and CP amino acid sequences. The Rep phylogenetic tree is rooted with representative sequences of circoviruses, whereas the CP phylogenetic tree is midpoint rooted. (b) Pairwise amino acid sequence identity matrix of the Rep and CP sequences. The representative sequences for each species are denoted with * next to the accession number.

Apart from avonheates virus SG2_28 (OM154938) and avonheates virus SG_28 (OM154945), which share 91.8% genome-wide pairwise identity, and Gas_1207 (OM154941) which belongs to the same species as AHEaBavV1 (KY405008), the other new bacilladnaviruses share 56.2–64.9% genome-wide pairwise identities to the previously classified bacilladnaviruses (Supplementary Data S1, available in the online Supplementary Material), suggesting that new taxa are needed for their classification. To extend the bacilladnavirus taxonomy, we searched the GenBank for other unclassified putative members of this family and identified three such bacilladnavirus genomes, namely Bacilladnaviridae sp. ctia23 (MH617734) and Bacilladnaviridae sp. ctdc18 (MH616678) from red snapper tissue [13] and Chaetoceros tenuissimus DNA virus SS12-43V (LC379168). Maximum likelihood phylogenetic analysis of the Rep sequences revealed four reasonably supported clades (>60% bootstrap support) and three singletons (Fig. 3a). Thus, we created four new genera to classify the new viruses and moved one species (accession no. KF133809) from *Protobacilladnavirus* to a new genus, *Seawadnavirus*.

The new genus names have been derived as follows:

1. *Aberdnavirus*: Derived from **aber** which is an estuary in Welsh
2. *Keisodnavirus*: Derived from **Keisō** (珪藻) which is a diatom in Japanese
3. *Puahadnavirus*: Derived from **pūaha**tanga which is an estuary in Māori
4. *Seawadnavirus*: Derived from **seawa**ter

**Fig. 4.**
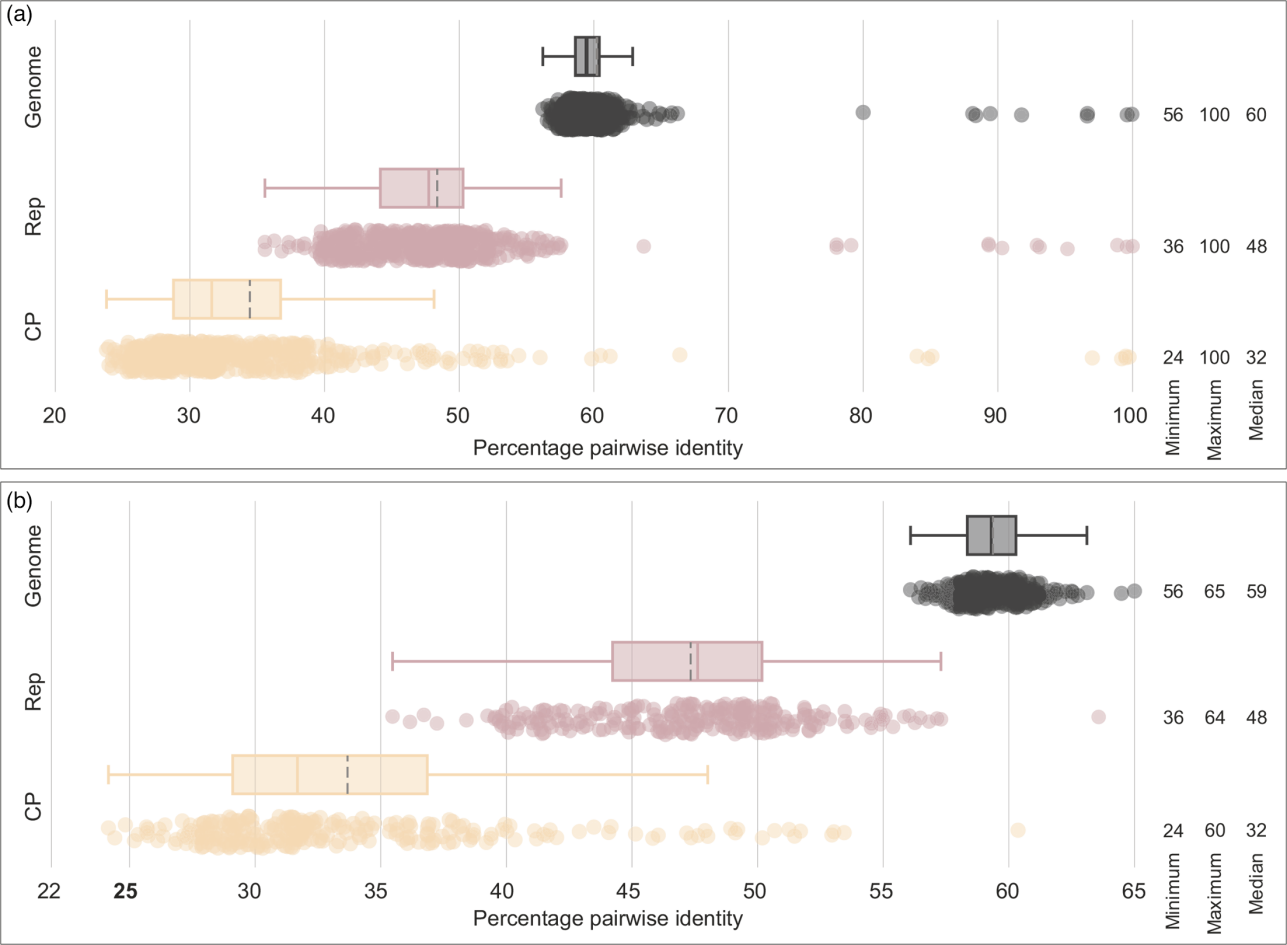
Boxplots showing the distribution of the genome nucleotide and Rep and CP amino acid pairwise identities. Pairwise identities were calculated for (a) all bacilladnavirus sequences (*n*=30) and (b) representative members from each species in the family *Bacilladnaviridae* (*n*=22). The range and median are provided on the right of the plot. The grey dotted line in the box plots represents the mean, whereas the solid line across the box represents the median. All pairwise comparisons were calculated using SDT v1.2 [26].

Genome as well as Rep and CP amino acid sequence comparisons showed that most members display 56–66% (median 59.5%), 35–63 % (median 47.7%) and 23–66 % (median 31.6) pairwise identities, respectively, with only a small fraction showing > 78 % (Fig. 4a). A 75% Rep amino acid pairwise identity as a species demarcation threshold was proposed for members of the genus *Protobacilladnavirus* [15] (because other genera were represented by singletons at the time). Based on the distribution of the pairwise identities of all 30 bacilladnavirus Rep amino acid sequences, we extend and apply the same demarcation criterion to all genera with more than one species. Notably, the species demarcation within *Bacilladnaviridae* differs from most other families within *Cressdnaviricota*, where genome-wide pairwise nucleotide identity threshold of 75–78 % is used instead (e.g. families *Circoviridae*, *Smacoviridae*, *Genomoviridae*, *Naryaviridae*, *Nenyaviridae* and *Vilyaviridae*) [37,40].

Sequences with accession numbers MH617734 and MH616678 represent two new species in the genus *Protobacilladnavirus*, and LC379168 represents a new species in a new genus, *Keisodnavirus*. The new bacilladnavirus sequences from Avon-Heathcote Estuary with accessions OM154943 and OM154949 represent two new species in the new genus *Seawadnavirus*; OM154940, OM154941, OM154942 and OM154947 represent new species in the genus *Kieseladnavirus*; and OM15494 is a member of the same species as the sequence with accession KY405008. Finally, the sequence with accession OM154938 (together with OM154945), OM154944, OM154948 and OM154950 represent four new species in the new genus *Puahadnavirus*, whereas OM154946 is the sole representative of a new genus *Aberdnavirus* (Table 1).

**Table 1.** Summary of the taxonomy of bacilladnaviruses with additional information Text in bold indicates new genera and species. Bold underlined text in the epithet notes highlights the source of the species epithet.

Genus	Accession	Virus name	Source	Country	Species	Epithet note	Reference
***Keisodnavirus***	LC379168	Chaetoceros tenuissimus DNA virus SS12-43V	*Chaetoceros tenuissimus*	Japan: Hiroshima Bay	***Keisodnavirus chaetenu***	***Chae*** *toceros **tenu**issimus*	–
*Protobacilladnavirus*	AB193315	Chaetoceros salsugineum DNA virus	*Chaetoceros salsugineum*	Japan: Fukuoka, Ariake Sea	***Protobacilladnavirus chasesal***	***Chae*** *toceros **sal**sugineum*	[5]
	MH617734	Bacilladnaviridae *sp*. isolate ctia23	Red snapper tissue	USA	***Protobacilladnavirus snap***	Red **snap**per	[13]
	AB844272	Chaetoceros *sp*. DNA virus 7	*Chaetoceros* sp. SS628-11	Japan: Hiroshima, Hiroshima Bay	***Protobacilladnavirus chaetoc***	***Chaetoc****eros sp*.	[8]
	AB553581	Chaetoceros lorenzianus DNA virus	*Chaetoceros lorenzianus*	Japan: Hiroshima, Itsukaichi Fishing Port	***Protobacilladnavirus chaelor***	***Chae*** *toceros **lor**enzianus*	[9]
	MH616678	Bacilladnaviridae *sp*. isolate ctdc18	Red snapper tissue	USA	***Protobacilladnavirus redsnap***	**Redsnap**per	[13]
	AB971660	Chaetoceros tenuissimus DNA virus type-II	*Chaetoceros tenuissimus* strain 2–10	Japan: Hiroshima, Hiroshima Bay	***Protobacilladnavirus tenuis***	*Chaetoceros **tenuis**simus*	[12]
	AB971659	Chaetoceros tenuissimus DNA virus type-II	*Chaetoceros tenuissimus* strain 2–10	Japan: Hiroshima, Hiroshima Bay	***Protobacilladnavirus tenuis***	*Chaetoceros **tenuis**simus*	[12]
	AB971658	Chaetoceros tenuissimus DNA virus type-II	*Chaetoceros tenuissimus* strain 2–10	Japan: Hiroshima, Hiroshima Bay	***Protobacilladnavirus tenuis***	*Chaetoceros **tenuis**simus*	[12]
	AB597949	Chaetoceros tenuissimus DNA virus	*Chaetoceros tenuissimus*	Japan: Fukuoka, Ariake Sound	***Protobacilladnavirus tenuis***	*Chaetoceros **tenuis**simus*	[7]
	KY405007	Amphibola crenata associated bacilladnavirus 2	*Amphibola crenata*	New Zealand (Aotearoa): Christchurch, Avon-Heathcote estuary	***Protobacilladnavirus hasleos***	***Hasle*** *a **os**trearia*	[2]
	MN229504	Haslea ostrearia associated bacilladnavirus	*Haslea ostrearia*	France: Bay of Bourgneuf	***Protobacilladnavirus hasleos***	***Hasle*** *a **os**trearia*	[10]
	MN229503	Haslea ostrearia associated bacilladnavirus	*Haslea ostrearia*	France: Bay of Bourgneuf	***Protobacilladnavirus hasleos***	***Hasle*** *a **os**trearia*	[10]
	KY405006	Amphibola crenata associated bacilladnavirus 1	*Amphibola crenata*	New Zealand (Aotearoa): Christchurch, Avon-Heathcote estuary	***Protobacilladnavirus mudflat***	**Mud-flat** snail common name for *Amphibola crenata*	[2]
***Puahadnavirus***	OM154950	Avonheates virus SG_479	Estuary benthic sediments	New Zealand (Aotearoa): Christchurch, Avon-Heathcote estuary	***Puahadnavirus kaisui***	Seawater in Japanese	This study
	OM154944	Avonheates virus SG_19	Estuary benthic sediments	New Zealand (Aotearoa): Christchurch, Avon-Heathcote estuary	***Puahadnavirus aber***	Estuary in Welsh	This study
	OM154948	Avonheates virus SG_146	Estuary benthic sediments	New Zealand (Aotearoa): Christchurch, Avon-Heathcote estuary	***Puahadnavirus inbhir***	Estuary in Scottish	This study
	OM154945	Avonheates virus SG_28	Estuary benthic sediments	New Zealand (Aotearoa): Christchurch, Avon-Heathcote estuary	***Puahadnavirus takutai***	Coast in Māori	This study
	OM154938	Avonheates virus SG2_28	Estuary benthic sediments	New Zealand (Aotearoa): Christchurch, Avon-Heathcote estuary	***Puahadnavirus takutai***	Coast in Māori	This study
***Seawadnavirus***	OM154949	Avonheates virus SG_154	Estuary benthic sediments	New Zealand (Aotearoa): Christchurch, Avon-Heathcote estuary	***Seawadnavirus gataifale***	Estuary/coast in Samoan	This study
	KF133809	Bacillariodnavirus LDMD-2013	Ocean water	–	***Seawadnavirus kuhtahan***	Seawater in Massachusetts	[14]
	OM154943	Avonheates virus SG_924	Estuary benthic sediments	New Zealand (Aotearoa): Christchurch, Avon-Heathcote estuary	***Seawadnavirus avonheat***	**Avon-Heat**hcote estuary	This study
*Kieseladnavirus*	OM154947	Avonheates virus SG_120	Estuary benthic sediments	New Zealand (Aotearoa): Christchurch, Avon-Heathcote estuary	***Kieseladnavirus katao***	Water in Māori	This study
	KY405008	Avon-Heathcote estuary associated bacilladnavirus	Estuary benthic sediments	New Zealand (Aotearoa): Christchurch, Avon-Heathcote estuary	***Kieseladnavirus ampcren***	***Amp*** *hibola **Cren**ata*	[2]
	OM154942	Avonheates virus SG_4_10	Estuary benthic sediments	New Zealand (Aotearoa): Christchurch, Avon-Heathcote estuary	***Kieseladnavirus titiko***	Amphibola crenata in Māori	This study
	OM154939	Avonheates virus Gas_102	*Amphibola crenata*	New Zealand (Aotearoa): Christchurch, Avon-Heathcote estuary	***Kieseladnavirus titiko***	Amphibola crenata in Māori	This study
	OM154941	Avonheates virus Gas_1207	*Amphibola crenata*	New Zealand (Aotearoa): Christchurch, Avon-Heathcote estuary	***Kieseladnavirus ampcren***	***Amp*** *hibola **Cren**ata*	This study
	OM154940	Avonheates virus Gas_1078	*Amphibola crenata*	New Zealand (Aotearoa): Christchurch, Avon-Heathcote estuary	***Kieseladnavirus karahue***	Common name for *Amphibola crenata* in Māori	This study
***Aberdnavirus***	OM154946	Avonheates virus SG_61	Estuary benthic sediments	New Zealand (Aotearoa): Christchurch, Avon-Heathcote estuary	***Aberdnavirus waitai***	Seawater in Māori	This study
*Diatodnavirus*	AB781089	Chaetoceros setoense DNA virus	*Chaetoceros setoensis*	Japan: Hiroshima Bay	***Diatodnavirus chaese***	***Chaet*** *oceros **se**toense*	[11]

To verify whether our species demarcation criterion is robust, we generated a dataset of representative sequences from each species. Analysis of the genome sequences, as well as the Rep and CP amino acid sequences of these representative sequences (*n*=22), shows that they share 56–65 % (median 59.4%), 35–64 % (median 47.7%) and 24-61% (median 31.8%), respectively (Fig. 4a), supporting the chosen species demarcation threshold.

To comply with the mandated binomial species naming format [41], we have renamed all existing species using binomial nomenclature with a freeform epithet. All epithets are either derivatives of host species or isolation sources using names in local languages of people that inhabit that region or settlers (Table 1).

### Increasing diversity of bacilladnaviruses provides insight into their evolution

Although, by definition, Rep sequences within each genus are monophyletic, this is not the case for the CPs. The tanglegram of the Rep and CP phylogenies (Fig. 3a) showed that the CPs of members of the genera *Protobacilladnavirus* and *Puahadnavirus* are polyphyletic, suggesting that recombination has likely played a role in their evolution, as is the case for many other ssDNA viruses [37,42,47]. Nevertheless, given the relatively high sequence conservation of the CPs within the family (≥ 24% identity in any pairwise comparison, Fig. 3b), the recombination appears to have taken place within the family, with no evidence of non-orthologous gene replacements in the currently known bacilladnaviruses. Notably, whereas bacilladnavirus Reps are generally more conserved than CP at the sequence level, in several cases, this pattern is inverted (Fig. 3b), further pointing to potential recombination events.

**Fig. 5.**
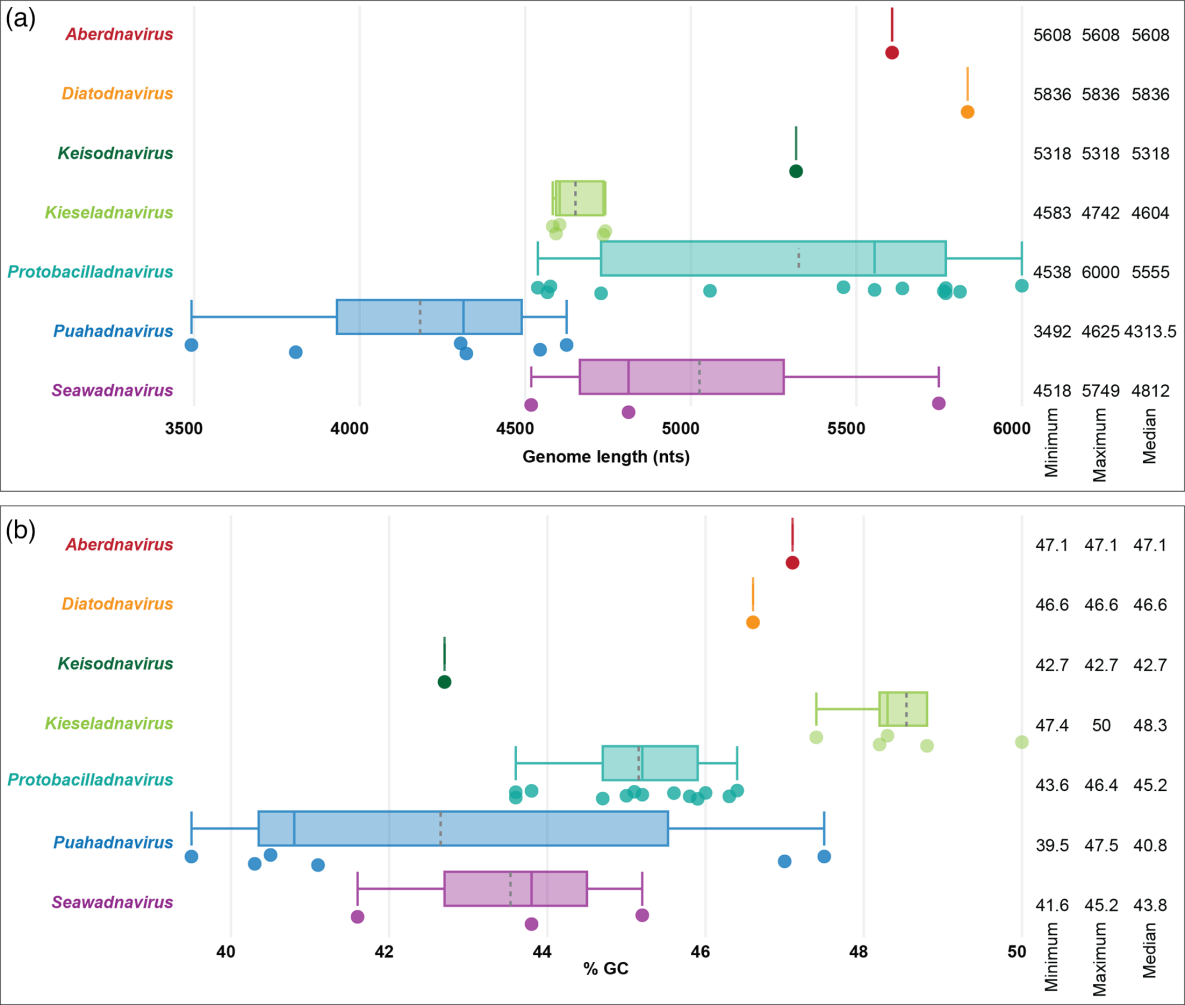
Boxplots showing the distribution of genome size (a) and percentage GC (b) in the genomes for members of each genus. The range and median are provided on the right of the plot, and the grey dotted line in the box plots represents the mean, whereas the solid line across the box represents the median.

**Fig. 6.**
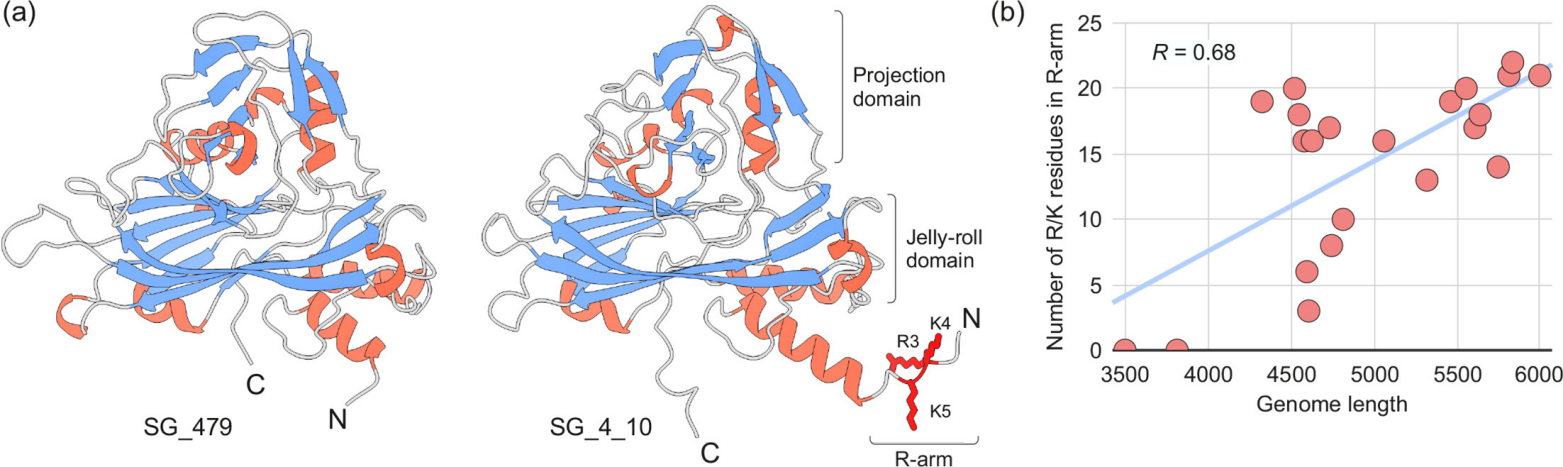
Capsid proteins of bacilladnaviruses. (a) Comparison of the modelled capsid protein structures of avonheates virus SG_479 (OM154950, species *Puahadnavirus kaisui*) and avonheates virus SG_4_10 (OM154942, species *Kieseladnavirus ampcren*), which lack the R-arm and contain a short R-arm, respectively. The *β*-strands are shown in blue and the *α*-helices in red. The arginine (r) and lysine (k) residues in the R-arm (N-terminus) are shown using stick representation. (b) Plot of the number of arginine (r) and lysine (k) in the R-arm of the bacilladnavirus capsid proteins relative to their genome size showing a positive Pearson correlation (*R*=0.68).

Analysis of the genome length distributions revealed a considerable variation among members of the family (Fig. 5a). Although genomes of bacilladnaviruses from most genera fall in the range of 4.5–6 kb, the largest genomes, those in the genus *Protobacilladnavirus* [up to 6000 nt for Chaetoceros salsugineum DNA virus (AB193315)], are nearly twice the size of certain viruses in the newly created genus *Puahadnavirus* [as small as 3492 nt for avonheates virus SG_479 (OM154950)]. Such size variation is likely to have consequences for the genome packaging density or the size and internal volume of the respective capsids. Comparison of the CPs encoded by viruses with the largest and smallest genomes, Chaetoceros salsugineum DNA virus and avonheates virus SG_479, respectively, showed that the former is 59 aa longer than the latter (392 vs. 333 aa). Analysis of the CP multiple sequence alignment showed that the extended N-terminal region in the CP of Chaetoceros salsugineum DNA virus accounts for most of the extra residues (Fig. S1). By contrast, the central part of the CP encompassing the jelly-roll and projection domains (Fig. 6a), which form the capsid shell and spike-like structures that point away from the capsid surface [2,4], was generally conserved, with only short insertions in some of the loops (Fig. S1). The largely unstructured N-terminal regions in the CP of Chaetoceros salsugineum DNA virus and other protobacilladnaviruses are enriched in positively charged amino acid residues and are oriented into the capsid lumen, where they are implicated in genome compaction. Similar N-terminal extensions, commonly known as the R-arm [[48]], are also found in the CPs of other eukaryotic ssDNA viruses, such as circoviruses [49] and anelloviruses [50,52]. Notably, the CP encoded by avonheates virus SG_19 (OM154944), another member of the *Puahadnavirus* genus with a relatively small genome (3807 nt), also lacks the R-arm. By contrast, the CPs of puahadnaviruses with genomes larger than 4 kb, similar to other bacilladnaviruses, such as avonheates virus SG_4_10 (OM154942), which is shown in Fig. 6(a) for comparison, contain the N-terminal regions enriched in arginine (R) and lysine (K) residues. Thus, the lack of the R-arm in the CPs of puahadnaviruses avonheates virus SG_479 and avonheates virus SG_19 suggests a relaxed necessity for the compaction of their smaller genomes. Consistently, the number of R and K residues in the R-arm (N-terminus) in the bacilladnavirus capsid proteins positively correlates with the genome length (Pearson correlation *R*=0.68, Fig. 6b). This result corroborates the conclusions of Requião *et al.* [48] that there is a positive correlation in the number of positively charged amino acids in the termini of capsid proteins of diverse DNA and RNA viruses with virus genome size. Indeed, given the conservation of the jelly-roll domains of bacilladnavirus CPs irrespective of their genome size (Fig. S1) the internal capsid volume of all bacilladnaviruses is expected to be similar, and hence, the accommodation of smaller genomes would require less compaction.

Notably, viruses in the *Puahadnavirus* genus displayed the largest variation in the %GC content (Fig. 5b). Although the exact hosts for bacilladnaviruses discovered by metagenomics remain unknown, the differences in %GC could reflect different host ranges for the corresponding viruses, as has been inferred for protist-infecting ssDNA viruses of the families *Naryaviridae*, *Nenyaviridae* and *Vilyaviridae* [45,53].

### Bacilladnaviruses encode a distinct phospholipase A1

Owing to their larger genomes afforded by T=3 capsids, the coding capacity of bacilladnaviruses is larger compared to other members of *Cressdnaviricota*. However, the functions encoded by bacilladnaviruses besides the CP and Rep remain unknown. The expanded bacilladnavirus genome diversity prompted us to reassess the functional annotation of hypothetical ORFs using sensitive profile–profile comparisons with HHsearch [27]. This analysis revealed a group of orthologs encoded by a subset of protobacilladnaviruses, diatodnavirus and aberdnavirus, which yielded multiple significant hits to phospholipase A1 (PLA1) (Table S1), a family of enzymes that catalyse the cleavage at the *sn*-1 position of phospholipids, forming a fatty acid and a lysophospholipid [54].

**Fig. 7.**
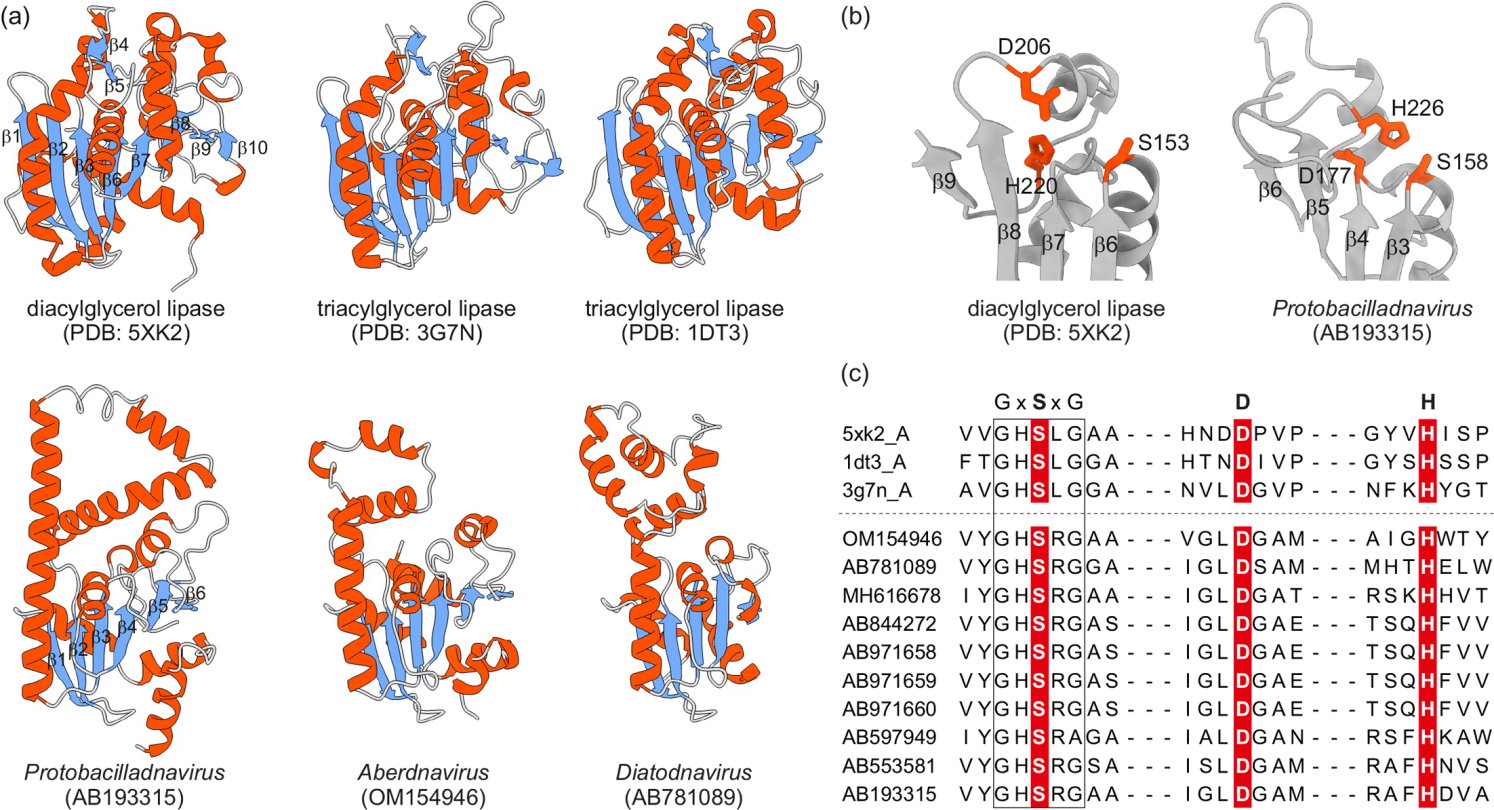
Cellular and viral PLA1 homologues. (a) Structures of cellular (top row) and bacilladnavirus (bottom row) PLA1 homologues. Bacilladnavirus structures were modelled using ColabFold [28], whereas cellular PLA1 X-ray structures were downloaded from PDB, and the corresponding accession numbers are provided in parentheses. Structures are coloured according to the secondary structure elements: *β*-strands, blue; *α*-helices, red; random coil, grey. *β*-strands are numbered for one cellular (*β*1–*β*10) and one viral (*β*1–*β*6) homolog (shown on the left). (b) Zoom-in on the core region encompassing the active site of cellular and viral PLA1 homologues. The experimentally determined cellular and predicted viral active site residues (red) are indicated using stick representation. (c) Alignment of the regions encompassing the putative active site residues (highlighted on red background) of bacilladnavirus PLA1 homologues. The catalytic Ser is located within a characteristic GxSxG motif (boxed), where x is any residue. Active site residues of the three cellular PLA1 proteins shown on the top were determined experimentally. Full-length alignment is provided in Fig. S3.

The HHsearch hits to PLA1 encompassed the signature GxSxG (x, any residue) motif, which includes the catalytic Ser residue of the PLA1 active site but does not extend to the catalytic Asp and His residues [54]. Thus, to further assess the possibility that bacilladnaviruses encode an active PLA1, we built structural models of the corresponding proteins representing the three genera using ColabFold [28] (Fig. S2). Searches queried with the obtained models against the protein structures available at the PDB database using DALI [[29]] produced best hits to PLA1 structures (*Z*-scores 7.2–8.2, Table S2). Comparison of the PLA1 structures and bacilladnavirus homologues indeed revealed structural similarities but also highlighted notable differences (Fig. 7a). In particular, the first (*β*1) and the last (*β*10) *β*-strand of the core 8-stranded *β*-sheet conserved in PLA1 members is missing in the bacilladnavirus proteins. Additionally, the characteristic *β*-hairpin (*β*4*–β*5) which follows *β*3 of the core *β*-sheet, is also lacking in bacilladnavirus homologues (Fig. 7a). Notably, however, although the GxSxG motif is present at equivalent positions (after *β*6 in cellular PLA1 and *β*3 of bacilladnavirus proteins), the location of the two other predicted active site residues, Asp and His, is radically different. Whereas in cellular PLA1 homologues, the catalytic Asp and His are present in the loop connecting *β*-strands *β*8 and *β*9 [55,57], bacilladnavirus proteins contain invariable Asp and His positions in the loops following *β*-strands *β*4 and *β*6, respectively (equivalent to β7 and β9 of cellular PLA1) (Fig. 6b). Importantly, despite being located within different loops in the bacilladnavirus proteins compared to the cellular PLA1 homologues, the three putative catalytic residues assume similar spatial positions (Fig. 7b). Furthermore, all three active site residues are invariable in the corresponding bacilladnavirus proteins (Fig. S3), suggesting that the latter are functional, catalytically active phospholipases. Notably, one protobacilladnavirus (MH617734) encodes an inactivated homologue of PLA1, which lacks all of the active site residues (Table S1).

The role of PLA1 homologues in the life cycle of bacilladnaviruses is unclear. Notably, phospholipases have been previously described in viruses with small RNA and DNA genomes. Among these, the best understood are PLA2 homologues encoded by certain ssDNA viruses of the *Parvoviridae* family [58]. In parvoviruses, PLA2 is expressed as an N-terminal extension of a minor capsid protein and is deployed during the entry process to liberate the internalized virions from the endosomes [59,60]. Subsequently, PLA2 domains were detected in RNA viruses, where they are also fused to the CP genes, suggesting a similar function as in parvoviruses [61]. The presence of PLA1 genes in viruses is less common, but not unprecedented. For instance, certain polinton-like viruses (*Aquintoviricetes*) and virophages (*Maviroviridae*) sporadically encode PLA1 homologues [62,63]. In the case of virophage mavirus, the virus-encoded PLA1 was detected in the virions [64], suggesting that it plays a role during virion entry, like in parvoviruses. Notably, SDS-PAGE analysis of the bacilladnavirus virions consistently revealed the presence of two major structural proteins [2,5, 7,10, 12]. However, the identity of the two proteins was not determined. Thus, although structural studies showed that the capsid shell is built from a single CP [4], it cannot be excluded that the PLA1 is present in the capsid lumen, as in the case of parvoviruses, and is involved in the virion liberation from the endosomes during entry. Alternatively or additionally, given that bacilladnaviruses lyse their diatom hosts at the end of the infection cycle, the PLA1 homologues could facilitate the virion egress. Notably, it has been recently demonstrated that the infection of *Chaetoceros tenuissimus* with protobacilladnavirus CtenDNAV resulted in a more statistically significant response in the lipidome than infection with a diatom RNA virus. In particular, virus-mediated lysis led to the production of chemical defence compounds called oxylipins, which are toxic chemical signalling molecules that are made by oxidizing fatty acids from membrane lipids [65]. We hypothesize that viral PLA1 could play a role in this process.

## Concluding remarks

Here, we describe the identification of three bacilladnavirus genomes from mud-flat snail/tītiko and ten from benthic sediments sampled from the Avon-Heathcote Estuary (Ihutai) in New Zealand (Aotearoa). We analysed these genomes along with other unclassified and classified bacilladnavirus genomes available in GenBank and based on the phylogeny of the Rep sequences, established four new genera (*Aberdnavirus*, *Keisodnavirus*, *Puahadnavirus* and *Seawadnavirus*) to classify the new viruses and move one species from the genus *Protobacilladnavirus* (accession no. KF133809) to the new genus, *Seawadnavirus*. The following are based on the 75% Rep amino acid sequence pairwise identity threshold we established:

1. One new species in the new genus *Aberdnavirus*
2. Two new species in the genus *Kieseladnavirus*
3. One new species in the new genus *Keisodnavirus*
4. Two new species in the genus *Protobacilladnavirus*
5. Five new species in the new genus *Puahadnavirus*
6. Two new species in the new genus *Seawadnavirus* and moved one species from *Protobacilladnavirus* to this genus

Furthermore, we renamed existing species and named the new species using a binomial format with a freeform epithet to comply with the mandated binomial species naming format. All taxonomic changes described above have been approved and officially ratified by the International Committee on Taxonomy of Viruses (ICTV) [16].

As more bacilladnaviruses are discovered, it is likely that the taxonomy will change. Thus, we discourage the community from using names of taxonomic ranks as part of the virus names, so that subsequent reassignment of a virus into a different taxon, as in the case of KF133809 which was moved from *Protobacilladnavirus* to genus *Seawadnavirus*, does not render the virus names misleading (e.g. protobacilladnavirus 1 becoming a member of genus *Seawadnavirus*). To avoid such situations, here we have named all 13 newly identified viruses as avonheates viruses (e.g. avonheates virus SG_61 that is deposited under OM154946), which stands for **Avon Heat**hcote **es**tuary followed by a unique code. Further, we also keep in mind that there is a clear distinction between a species name and a virus name; see Zerbini *et al*. [66].

All members of the community studying bacilladnaviruses are encouraged to submit taxonomy proposals for new species and genera to the ICTV (see https://ictv.global/). The Virus Metadata Resource (https://ictv.global/vmr) is updated annually and is an excellent resource for metadata on species and representative members of each species.

Finally, the analysis of the expanded diversity of bacilladnavirus genome and protein sequences provided new insights into the biology and evolution of this understudied group of viruses. Comparison of the bacilladnavirus CP sequences suggests that the R-arm-mediated compaction is only required for encapsidation of the larger genomes, whereas smaller genomes can be packaged in the absence of the R-arm subdomain. The presence of a highly derived PLA1 in members of three *Bacilladnaviridae* genera suggests a mechanism of penetration through the cellular membranes during the entry and/or egress stages. Notably, bacilladnaviruses, which do not encode recognizable PLA1 homologues, carry hypothetical genes at the equivalent position. We hypothesize that the products of these genes play analogous roles during the infection. Testing the activities of the divergent bacilladnavirus PLA1 homologues, as well as those of their positional counterparts, appears to be a promising research direction that can uncover enzymes with new functionalities and biotechnological potential.

## supplementary material

10.1099/jgv.0.002084 Supplementary Material 1.

10.1099/jgv.0.002084 Supplementary Data Sheet 1.

## Abbreviations

CP: capsid protein
ICTV: International Committee on Taxonomy of Viruses
PDB: Protein Data Bank
PLA1: phospholipase A1
RCA: rolling circle amplification
Rep: replication-associated protein
