# Supplementary material for: Bacilladnaviridae: refined taxonomy and new insights into the biology and evolution of diatom-infecting DNA viruses
Source: J Gen Virol. 2025 Mar 12;106(3):002084. doi: 10.1099/jgv.0.002084 (PMC11903649; doi:10.1099/jgv.0.002084)
Supplement: Supplementary Material 1. [file jgv-106-02084-s001.pdf]

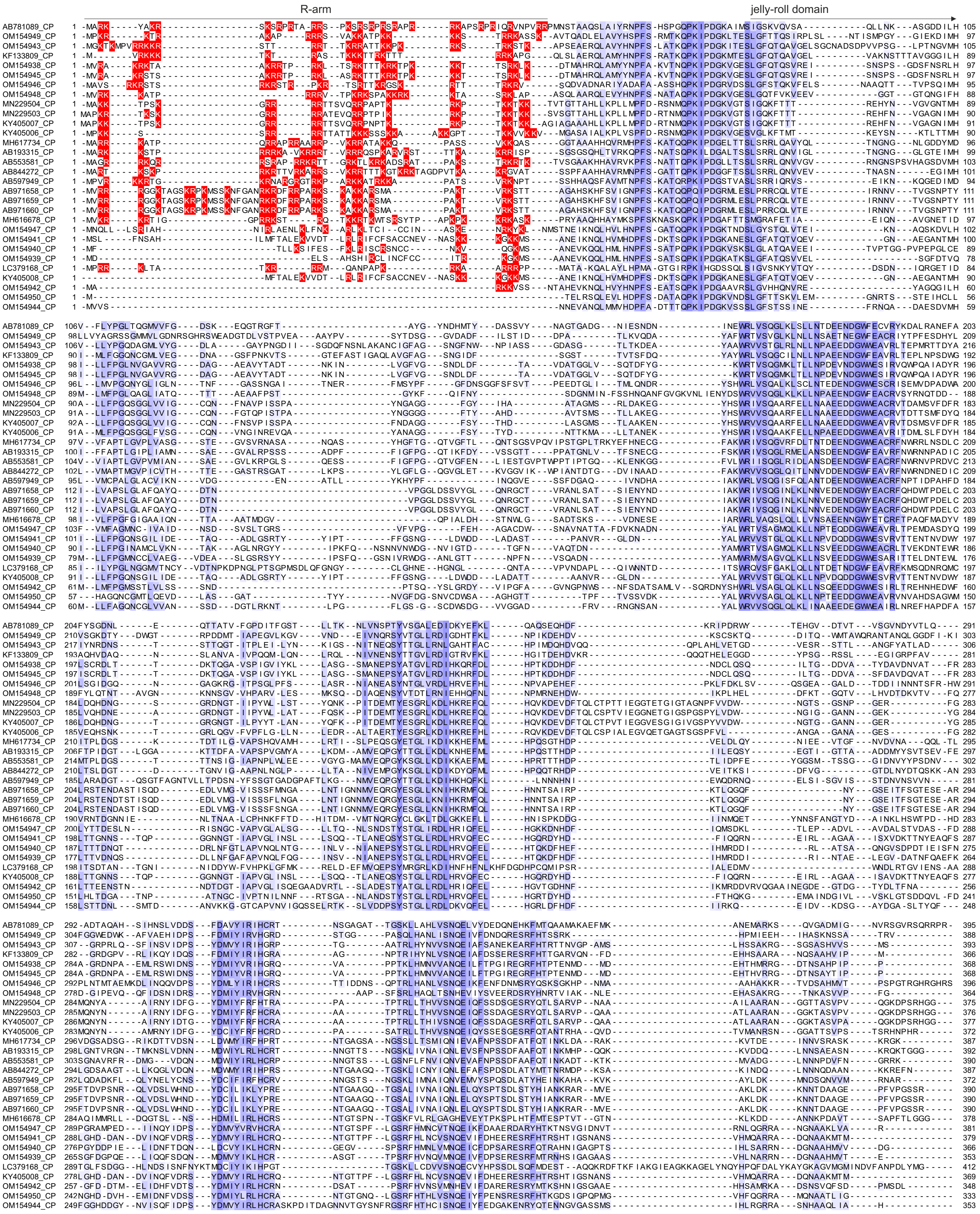

Supplementary Figure 1: Multiple sequence alignment of the bacilladnavirus capsid proteins. The sequences were aligned using PROMALS3D [24]. The alignment is colored according to conservation (Blosum62 matrix) and visualized with Jalview [25]. Arg and Lys residues in the R-arm are highlighted with red background. The beginning of the jelly-roll domain is indicated above the alignment.

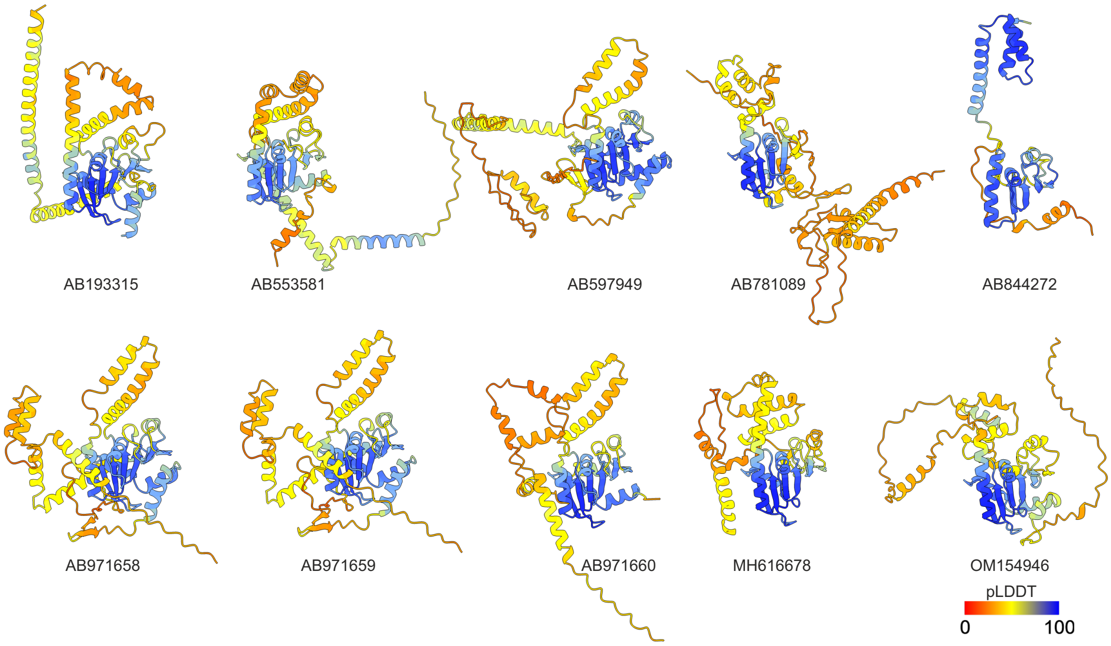

Supplementary Figure 2: Structural models of the putative PLA1 homologs encoded by bacilladnaviruses. The models are colored according to their pLDDT values, with the color key provided at the bottom right corner.

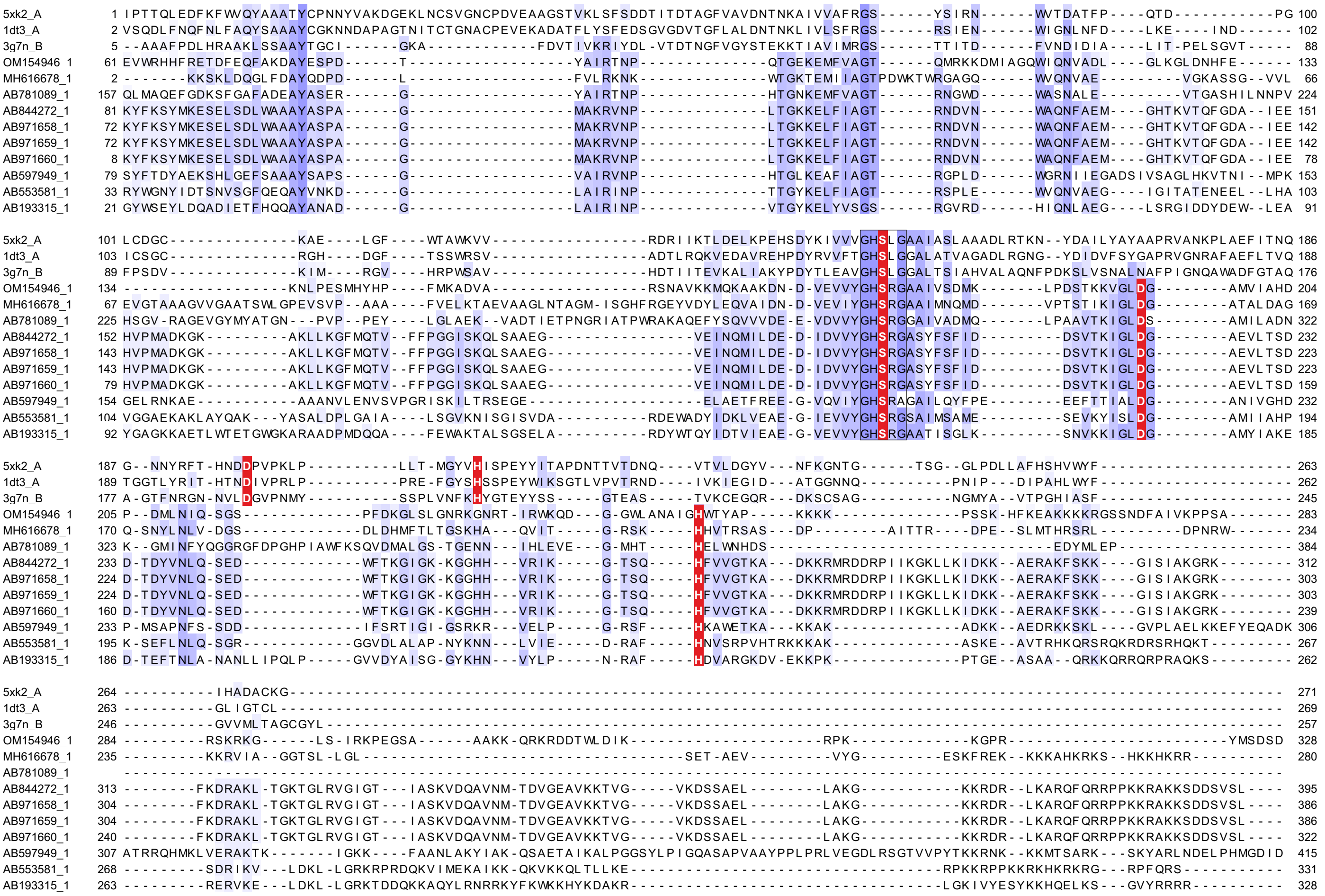

Supplementary Figure 3: Multiple sequence alignment of the PLA1 homologs from bacilladnaviruses and cellular organisms. The top three sequences represent cellular PLA1 homologs with available 3D structures and are identified with the corresponding PDB accession numbers. The alignment is colored according to conservation (Blosum62 matrix) and visualized with Jalview [25]. The catalytic triad is highlighted with red background.

Supplementary table 1. Results of the HHsearch analysis of the previously unannotated bacilladnavirus proteins.

Virus protein	Annotation	HHsearch profile	HHsearch probability
AB193315_1	Phospholipase A1	7X0D_B Phospholipase A1; Phospholipase A1, HYDROLASE; HET: SO4; 2.3972515576A [Capsicum annuum]	96.42
AB553581_1	Phospholipase A1	7X0D_B Phospholipase A1; Phospholipase A1, HYDROLASE; HET: SO4; 2.3972515576A [Capsicum annuum]	95.48
AB597949_1	Phospholipase A1	7X0D_B Phospholipase A1; Phospholipase A1, HYDROLASE; HET: SO4; 2.3972515576A [Capsicum annuum]	93.68
AB781089_1	Phospholipase A1	7X0D_B Phospholipase A1; Phospholipase A1, HYDROLASE; HET: SO4; 2.3972515576A [Capsicum annuum]	95.51
AB844272_1	Phospholipase A1	7OTS_A Monoacylglycerol lipase ABHD6; alpha/beta-Hydrolase domain containing 6 2-arachidonoylglycerol hydrolase monoacylglycerol lipase ABHD6 endocannabinoid system 2-AG; HET: OLA, SO4, BOG, GOL; 1.792A [Homo sapiens]	98.97
AB971658_1	Phospholipase A1	7X0D_B Phospholipase A1; Phospholipase A1, HYDROLASE; HET: SO4; 2.3972515576A [Capsicum annuum]	94.94
AB971659_1	Phospholipase A1	7X0D_B Phospholipase A1; Phospholipase A1, HYDROLASE; HET: SO4; 2.3972515576A [Capsicum annuum]	94.94
AB971660_1	Phospholipase A1	7X0D_B Phospholipase A1; Phospholipase A1, HYDROLASE; HET: SO4; 2.3972515576A [Capsicum annuum]	95.3
MH616678_1	Phospholipase A1	7X0D_B Phospholipase A1; Phospholipase A1, HYDROLASE; HET: SO4; 2.3972515576A [Capsicum annuum]	95.07
OM154946_1	Phospholipase A1	7X0D_B Phospholipase A1; Phospholipase A1, HYDROLASE; HET: SO4; 2.3972515576A [Capsicum annuum]	95.83
MH617734_1	Phospholipase A1 (inactivated ortholog of AB781089_1)	P0DOK3 VP1_CDDV1 Viral protein 1 OS=Chaetoceros diatodnavirus 1 OX=2169869 PE=4 SV=1	96.89

Supplementary table 2. Results of the DALI searches queried with the bacilladnavirus PLA1 homologs against the PDB database.

Virus protein	pLDDT	DALI hit in PDB	Z-score
AB193315_1	73.1	5xk2-A mono- and diacylglycerol lipase from *Aspergillus oryzae*	8.2
AB553581_1	68.2	7xey-B lipase-like protein PAD4	8
AB597949_1	67.9	7xey-B lipase-like protein PAD4	7.4
AB781089_1	55.7	2qua-A lipase LipA from *Serratia marcescens*	7.5
AB844272_1	79.6	4fle-A esterase YqiA from *Yersinia enterocolitica*	7.2
AB971658_1	67.2	7x0d-A phospholipase A1 from *Capsicum annuum*	7.8
AB971659_1	67.1	7x0d-A phospholipase A1 from *Capsicum annuum*	7.9
AB971660_1	66.3	6nkc-B lipase Lip_vut1 from goat rumen metagenome	7.9
MH616678_1	68	1uwc-A feruloyl esterase from *Aspergillus niger*	8
OM154946_1	66.6	1dt3-A lipase from *Thermomyces lanuginosa*	7.8
